# Indocyanine green-assisted lymphography for intraoperative chyle leak prevention during esophageal cancer surgery: a systematic review of the literature

**DOI:** 10.3389/fonc.2026.1741834

**Published:** 2026-03-03

**Authors:** Francesco Puccetti, Francesco Saverio Candiloro, Lorenzo Cinelli, Silvia Battaglia, Lorenzo Gozzini, Ugo Elmore, Riccardo Rosati

**Affiliations:** 1 Faculty of Medicine, Vita-Salute San Raffaele University, Milan, Italy; 2 Department of Gastrointestinal Surgery, Istituto di Ricovero e Cura a Carattere Scientifico (IRCCS) San Raffaele Scientific Institute, Milan, Italy

**Keywords:** chyle leakage, esophageal cancer, esophagectomy, fluorescence-guided surgery, minimally invasive surgery

## Abstract

**Background and aim:**

Chyle leakage (CL) is a potentially life-threatening complication, severely impacting postoperative recovery after esophageal cancer resections. Indocyanine green-assisted lymphography (ICG-Lg) seems to provide a fluorescent visualization of the thoracic duct (TD), although the optimal approach for CL prevention has not been defined.

**Methods:**

This study was designed as a systematic review and included either randomized or observational reports regarding ICG-Lg during esophageal cancer resections. The literature search was conducted on PubMed, Embase, and Scopus databases, and original articles combining ICG-Lg during esophageal cancer resections were selected. The rate of TD visualization was primarily investigated, while secondary outcomes included procedural complications, CL incidence, length of stay, and lymph node harvest (LNH). The review was registered on PROSPERO (CRD42025638309) and was performed according to PRISMA guidelines.

**Results:**

Thirteen non-randomized articles were selected, including 1218 patients undergoing surgery for esophageal cancer. MINORS study quality assessment showed moderate scores (74.5%). The TD was correctly visualized in 95.4% and generally preserved (68.1%) for CL prevention. Procedural complications were negligible (0.1%), and CL occurrence was significantly lower after ICG-Lg (1.4 vs 5.4%, P<0.001). The studies demonstrated a shorter LOS (OR -0.13, 95% CI -0.30 to 0.04, P = 0.273) and a significantly higher LNH (OR 0.40, 95% CI -0.20 to 1.00, P<0.001) after ICG-Lg.

**Conclusions:**

Although intraoperative ICG-Lg provides a safe and effective TD visualization during esophagectomy, minimization of postoperative CL and maintenance of extensive lymph node dissection depends on the surgical strategy. Randomized trials should be specifically designed to identify surgical determinants of CL prevention in esophageal cancer surgery.

**Systematic Review Registration:**

https://www.crd.york.ac.uk/prospero/, identifier CRD42025638309.

## Introduction

Chyle leak (CL) after esophagectomy accounts for 4.6% of postoperative complications, leading to higher rates of respiratory insufficiency and 90-day mortality (1). The intraoperative injury of the thoracic duct (TD) is the most common cause of CL, which occurs during intrathoracic esophageal dissection and lymphadenectomy and appears as a milky discharge from chest drainage upon the initiation of enteric feeding. Postoperative diagnosis can be accurately made through pleural fluid analysis demonstrating triglyceride level >110 mg/dL and/or chylomicrons in pleural fluid (2). Patients with CL are more likely to experience pulmonary complications entailing extended length of hospital stay (LOS) or an increase in the level of care, due to higher rates of atelectasis, pneumonia, or need for re-intubation (3). Recently, stepwise complication management has been established as the most appropriate treatment for CL depending on the severity of clinical presentation. Complication management involves a stepwise approach that includes multiple therapeutic options of consecutive invasiveness, such as dietary interventions (e.g., intake of a low-fat diet, medium-chain triglycerides enteral nutrition, total parenteral nutrition), administration of medications (Somatostatin analogs like Octreotide or alpha-agonists like Etilefrine), interventional radiology procedures (e.g., intranodal lymphography and Lipiodol^®^ embolization), and reoperation for leak repair (4). Given the uncertain outcomes following CL treatments, several high-volume esophageal centers developed and relied on different types of surgical prevention, although their efficacy remains controversial. Traditionally, control strategies were restricted to pre-emptive TD preservation or ligation during the intrathoracic dissection of the esophagus, although poor visualization and possible anatomical variations may limit the accuracy of surgical maneuvers. Conversely, both issues have been overcome by using intraoperative near-infrared (NIR) lymphography with indocyanine green (ICG), a fluorescent dye with amphiphilic properties that facilitates solubility in biological fluids and bioavailability. Excitation and emission wavelengths of ICG are located in the NIR range (780 to 830nm), making it suitable for detection by dedicated NIR-camera systems (5). However, the literature has not previously reported on procedural standardization for ICG-mediated lymphography (ICG-Lg) for TD visualization, including significant heterogeneity has been described in terms of dosages, administration routes, and injection timing (6).

The rationale of this systematic review is that intraoperative ICG-Lg can provide an effective and safe TD visualization, potentially leading to CL prevention and adequate oncological resections for esophageal cancer. Therefore, this study aims to assess the ICG-Lg effectiveness over the literature, in esophageal cancer surgery, and highlight operative characteristics and outcomes regarding TD visualization, CL prevention, and procedural complications.

## Materials and methods

This systematic review of the literature was conducted and reported in compliance with the PRISMA guidelines (PRISMA checklist in the **Supplementary Material 1**) and was recorded in PROSPERO database (registration number: CRD42025638309) (7, 8).

### Study design and search strategy

The present systematic review aimed to gather all ICG-based intraoperative techniques for CL prevention in esophageal cancer surgery. The literature search was performed by probing main search engines (i.e., PubMed, Embase, and Scopus database) with detailed queries based on PICO (Population, Intervention, Comparison, and Outcome) strategy and specific machine syntax (**Supplementary Material 2**) (9). All search findings were independently conducted by two authors (F.C. and F.P.) in January 2025. A first-round selection was performed through Rayyan-assisted abstract screening, and a full-text examination was consecutively performed according to the inclusion criteria. Any conflictual assessment was further discussed with another author (L.C.) to reach an agreement for article selection. The literature search was performed from the date of the earliest ICG-Lg description (December 2020) to January 2025 (10). Inclusion criteria were original articles reporting ICG-based techniques to visualize the TD during esophageal cancer surgery, regardless of the choice for surgical manipulation or the combination with neoadjuvant therapy. Exclusion criteria involved operations other than esophagectomy, ICG-Lg for postoperative complications, or any designs other than cohort analyses.

### Study quality and bias assessment

The appraisal of search findings was performed according to the study design, and the Methodological Index of Non-Randomized Studies (MINORS) was primarily used for either comparative or non-comparative cohort studies (11). MINORS assessment is structured in 12 methodological items, which range from 0 (not reported) to 2 (reported and adequate), composing the comprehensive quality score up to the highest score of 16 for non-comparative or 24 for comparative studies (**Supplementary Material 3**).

### Outcome definition and data analysis

Data extraction captured study features (i.e., first author, year and country of publication, study design), baseline population characteristics (i.e., size, sex ratio, median age and mean Body Mass Index), surgical and ICG-Lg details (i.e., primary disease and operation, neoadjuvant therapy, ICG dose and administration route, pre-emptive procedure for CL, mean observation and operative time), and postoperative results (i.e., success rate and time for TD visualization, procedural complications of ICG-Lg, lymph node harvest (LNH), incidence of postoperative CL, LOS). The rate of TD visualization after ICG-Lg was the primary outcome, while the occurrence of procedural complications and postoperative CL was secondarily evaluated. Lymph node harvest and LOS were also included in secondary outcomes. Descriptive analysis was performed to condense the comparative studies’ main findings. Inferential statistics was set up according to data availability and variable types. Pooled outcome results were calculated using random-effects models to minimize potential biases due to the variety of effect estimations (odds ratios, OR) between studies (12). Results were represented through forest plots, with 95% CI and a p-value <0.05 for statistical significance. Study heterogeneity was assessed and reported with I^2^ index (i.e., mild and significant heterogeneity accounted for <30% and >50%, respectively). Analyses were performed using Open Meta-Analyst software (Version 10.10) by the Center for Evidence-Based Medicine of Brown University (13).

## Results

### Search results and study population

**Figure 1:**
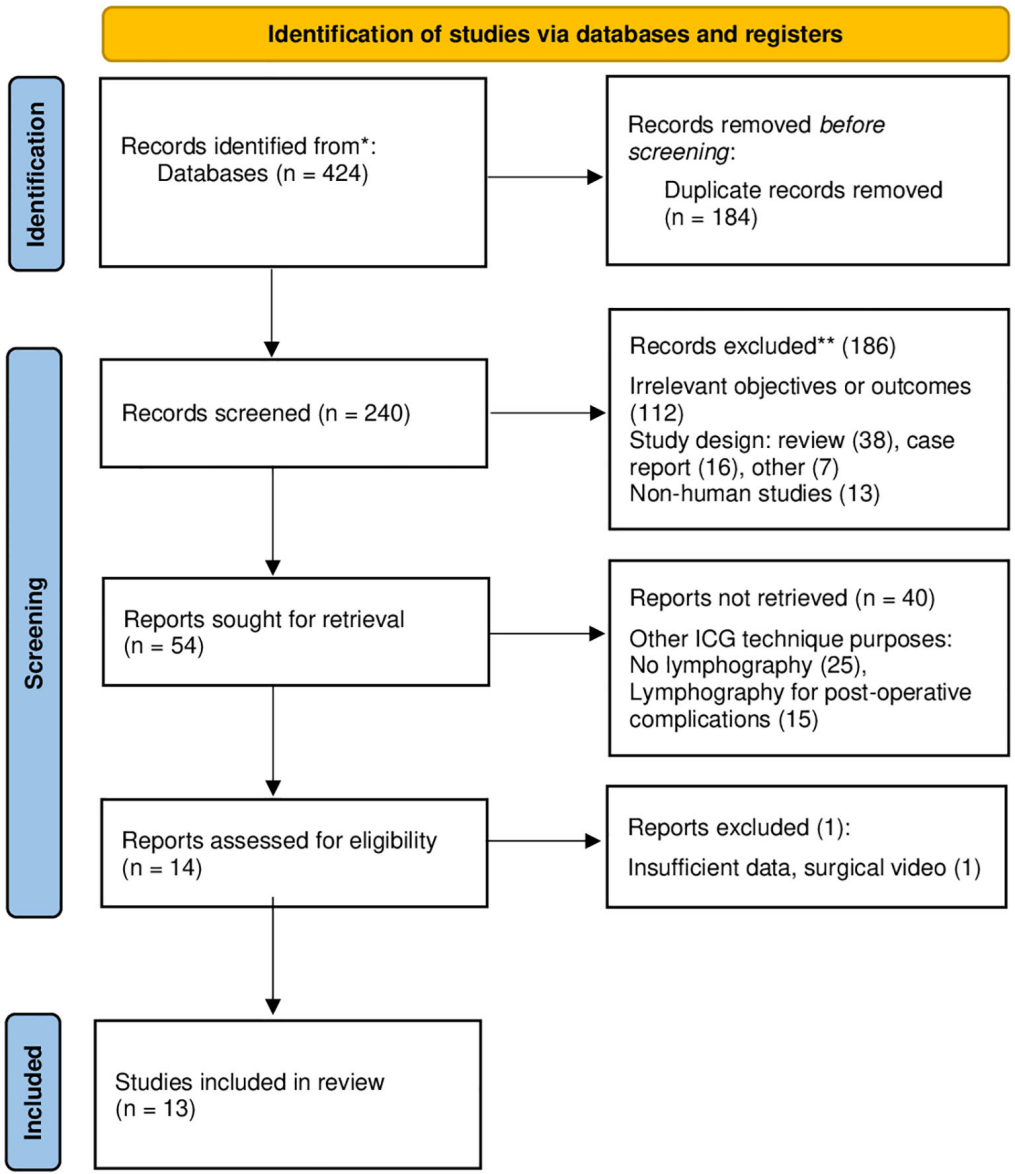
PRISMA flow diagram of search strategy.

The search strategy shrank eligibility for inclusion in the present analysis from 424 to 13 results (10, 14–25), including 1218 patients undergoing esophageal cancer surgery (**Figure 1**). The literature search reported exclusively single-center non-randomized trials, gathering multiple types of surgical techniques from esophageal centers worldwide (**Table 1**). According to the specific study design, all selected articles were evaluated through the MINORS quality criteria (**Supplementary Material 2**) and demonstrated a mean quality score as high as 74.5%, reporting slightly better accuracy in comparative than non-comparative studies (77.5 vs 72.7%, respectively). Intraoperative ICG-Lg was performed in 703 patients (57.7%) in combination with different levels of minimally invasive surgery: video-assisted thoracoscopic surgery (VATS, 86.8%), robot-assisted thoracoscopic surgery (RATS, 10.5%), and open esophagectomy (Op, 2.7%). Patients submitted to ICG-Lg were predominantly male (72.8%), and underwent two-field esophagectomy (62.3%) for squamous cell carcinoma (65.0%). Neoadjuvant therapy was administered in 442 patients (62.9%), who received chemo or chemo-radiotherapy (25.4 and 36.4%, respectively).

**Table 1:** Baseline study characteristics.

Study	Study design	Period	Country	Study population^a^	Sex (M/F)	Age	BMI	Esophagectomy fields (%)	Chest stage	Operating time (min)	Disease (%)	Neoadjuvant (%)
Ji, et al. 2024 (14)	CC	Jan 2019 – Aug 2023	China	179/354	140/39	66	n.a.	2 (15.1), 3 (84.9)	VATS	200 (±49)	SCC (93.3), AC (5.0), Other (1.7)	n.s. (21.8)
Mahmoodzadeh, et al. 2024 (15)	CC	Sep 2020 – Jun 2022	Iran	18/36	8/10	56.4	23.4	2 (100)	VATS	366.67	SCC (66.7), AC (33.3)	CRT (100)
Ao, et al. 2024 (16)	CC	Sep 2017 - Sep 2019	China	59/118	47/12	63 (40-77)	21.7	2 (93.2), 3 (6.8)	VATS	180 (140-420)	SCC (96.6), Other (3.4)	0
Puccetti, et al. 2024 (17)	CC	Jan 2018 - Aug 2023	Italy	151/320	126/25	65 (57-71)	25.5	2 (100)	VATS	273 (251-297)	SCC (21.2), AC (78.8)	CT (58.9), CRT (33.8), None (7.3)
Somashekhar, et al. 2024 (18)	PC	Jan 2020 - Jul 2022	India	50/50	32/18	47.4 (±6.7)	23.2	3 (100)	RATS	321.13 (±13.75)	SCC (56.0), AC (44.0)	CRT (56.0), CT (44.0)
Aw, et al. 2023 (19)	CC	Jul 2019 - Jul 2022	Canada	12/105	8/4	74 (66-76)	26.7	2 (83.3), 3 (16.7)	VATS, Open	n.a.	SCC (16.7), AC (8.3)	CRT (91.7), CT (8.3)
Thammineedi, et al. 2023 (20)	RC	Oct 2020 - Jan 2023	India	99/99	43/56	55 (22-77)	n.a.	2 (100)	VATS	450 (300-660)	SCC (79.8), AC (19.2), Other (1.0)	CRT (87.9), CT (11.1), None (1.0)
Barbato, et al. 2022 (21)	PC	Oct 2019 - Oct 2021	Italy	18/18	15/18	67.3 (48-83)	n.a.	2 (100)	RATS	n.a.	AC (100)	CT (100)
Barnes, et al. 2022 (22)	PC	Dec 2017 – Aug 2018	UK	20/20	16/4	62 (37-78)	25.7	2 (100)	VATS, Open	n.a.	n.a.	n.a.
Yang, et al. 2022 (23)	PC	Dec 2020 - Dec 2021	China	41/41	35/6	64 (58-67)	21.6	2 (78.0), 3 (22.0)	VATS	n.a.	SCC (90.2), AC (4.9), Other (4.9)	CT (31.7)
Tokumaru, et al. 2022 (24)	RC	Jun 2020 - Jan 2022	Japan	16/16	14/2	66 (22-79)	23.4	3 (100)	VATS	383.5 (221-506)	SCC (62.5), AC (31.3), Other (6.2)	CT (81.3), n.s. (6.2), None (12.5)
Varshney, et al. 2022 (25)	RC	Jan 2020 - Dec 2021	India	21/21	13/8	54	20.6	3 (100)	VATS, RATS	n.a.	SCC (100)	CRT (42.9), CT (57.1)
Vecchiato, et al. 2020 (10)	PC	Jul 2018 - Jan 2019	Italy	19/20	15/4	67.8	n.a.	2 (42.1), 3 (52.6)	VATS	258	SCC (60.0), AC (40.0)	CRT (100)

^a^ICG group / Entire population.

n.a., Not applicable; PC, Prospective cohort study; RC, Retrospective cohort study; CC, Combined cohort study; VATS, Video-assisted thoracoscopic esophagectomy (VATS); RATS, Robot-assisted thoracoscopic esophagectomy; Op, Open esophagectomy; SCC, Squamous cell cancer; AC, Adenocarcinoma; CT, Chemotherapy; RT, Radiotherapy; CRT, Chemoradiotherapy; n.s., Not stated.

### Outcomes analysis

**Figure 2:**
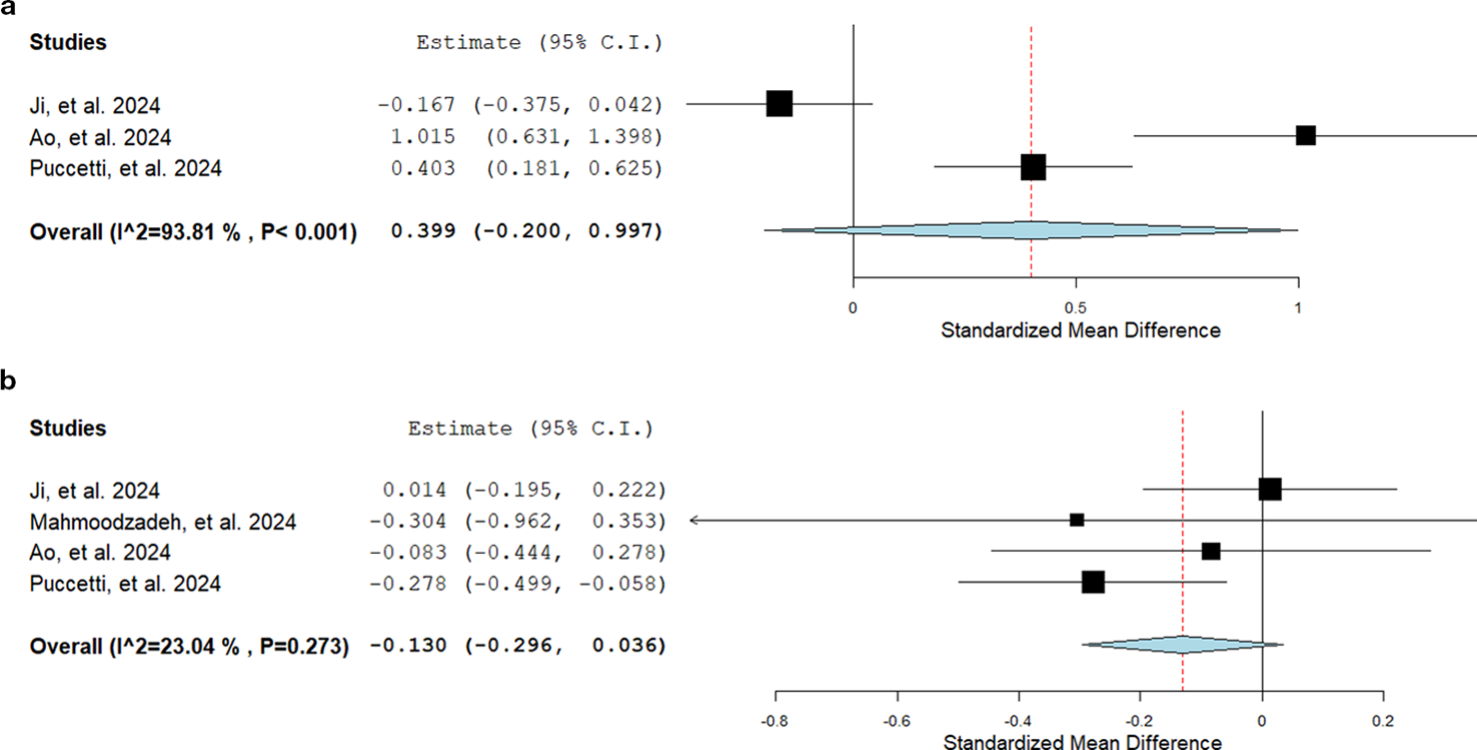
Forest plot analyses for lymph node harvest **(a)** and hospital stay **(b)**. (A) Lymph node harvest (LNH) analysis includes three (out of five) comparative studies. The three studies reported different surgical strategies following the thoracic duct identification: preservation (Ji, et al), preservation with NIR-guided lymphadenectomy (Ao, et al), and ligation and dissection along with loco-regional lymph node stations (Puccetti, et al). (B) Length of hospital stay (LOS) analysis includes four (out of five) comparative studies.

Regardless of technical variations among selected studies, the overall rate of TD visualization following ICG-Lg was as high as 95.4%. The administration dosage and route appeared to be arbitrarily chosen by individual centers, even though the primary injection site was the inguinal lymph nodes (8 studies, 513 (73.0%) pts) (10, 14, 15, 17–20, 25). The ICG tracer was variably administered through weight-based (from 0.2 to 0.5 mg/kg) or fixed dosages (from 2 to 25 mg), reporting considerably different practices. However, no procedural complications were reported in the entire study population, with the exception of one case of temporary skin discoloration at the ICG injection site (0.1%). The TD preservation following the fluorescent visualization was the most frequent surgical strategy for CL prevention (11 studies, (68.1%) patients) (10, 14–16, 18–20, 22–25), although no general agreement defined a standardized intraoperative management. Intraoperative CL occurs in 23 patients (3.3%), demonstrating the failure rate of TD preservation and justifying the need for ligation in case of early evidence of iatrogenic injury. Given the high heterogeneity of surgical practice, this analysis could not measure the pre-emptive strategy-weighted impact on postoperative CL (**Table 2**). The postoperative CL occurred in 10 (1.4%) patients who received intraoperative ICG-Lg, while 28 (5.4%) cases developed the complication following esophagectomy without fluorescence (P<0.001). Comparative studies demonstrated a shorter LOS (odds ratios [OR] -0.13, 95% confidence interval [CI] -0.30 to 0.04, P = 0.273) and a significantly higher LNH (OR 0.40, 95% CI -0.20 to 1.00, P<0.001), although the high study heterogeneity did not allow a comprehensive elucidation of all clinical determinants for improvements after ICG-Lg (**Figure 2**).

**Table 2:** Procedural features and outcomes of ICG-Lg.

Study	ICG	Adm. dosage	Adm. route (%)	Fluorescence rate (%)	Time^a^ to fluorescence	Prevention of CL (%)	Intraop. CL (%)	Postop. CL (%)	Adverse reaction	LOS^b^
Ji, et al. 2024 (14)	Diagnogreen	0.5 mg/kg	ILN (179)	177 (98.9)	n.a.	Preservation (91.6) Ligation (8.4)	9 (5.0)	0	0	13.7 (±7.5)
Mahmoodzadeh, et al. 2024 (15)	n.a.	10 mg	ILN (18)	18 (100)	n.a.	Preservation (94.4) Ligation (5.6)	1 (5.5)	0	0	10.8 (±6.0)
Ao, et al. 2024 (16)	DYPh Co. ^c^	0.5–2.5 mg	SM (59)	59 (100)	n.a.	Preservation (100)	n.a.	0	n.a.	10 (7–62)
Puccetti, et al. 2024 (17)	Verdye	25 mg	ILN (151)	149 (98.7)	15-30	Ligation and resection (100)	n.a.	7 (4.6)	n.a.	9 (8–15)
Somashekhar, et al. 2024 (18)	n.a.	1 ml ^d^	ILN (25)	25 (100)	20 (16-20)	Preservation (100)	0	0	n.a.	n.a.
			WS (25)	18 (72.0)	45					
Aw, et al. 2023 (19)	Spy Agent Green ICG	5 mg	ILN (2) SBM (9) WS (1)	6 (50.0)	n.a.	Preservation (100)	0	1 (8.3)	n.a.	10
Thammineedi, et al. 2023 (20)	Aurogreen	5 mg	ILN (99)	93 (93.9)	60 (30-330)	Preservation (80.8) Ligation (19.2)	n.a.	0	0	7 (5-15)
Barbato, et al. 2022 (21)	n.a.	0.5 mg/kg	SC (18)	18 (100)	20 (18-24) ^e^	Ligation and resection (100)	1 (5.5)	0	0	n.a.
Barnes, et al. 2022 (22)	n.a.	6.25 mg	FJ (3)	0 (0)	84 (10-185)	Preservation (70.0) Ligation (30.0)	6 (30.0)	1 (5.0)	0	10 (7-40)
		3.75-5 mg	SBM (17)	16 (94.1)						
Yang, et al. 2022 (23)	DYPh Co. ^c^	20 mg	SC (41)	38 (92.7)	30	Preservation (92.7) Ligation (7.3)	3 (7.3)	0	0	n.a.
Tokumaru, et al. 2022 (24)	Diagnogreen	0.2–0.5 mg/kg	SC (16)	14 (87.5)	119 (56-199)	Preservation (68.7) Ligation (31.3)	2 (12.5)	1 (6.25)	1 (6.25)	n.a.
Varshney, et al. 2022 (25)	Aurogreen	2-3 mg	ILN (21)	21 (100)	35 (30-35)	Preservation (85.7) Ligation (14.3)	1 (4.8)	0	0	6
Vecchiato, et al. 2020 (10)	Verdye	0.5 mg/kg	ILN (18)	18 (100)	52.7 (35-80)	Preservation (84.2) Ligation (15.8)	0	0	0	10
			WS (1)	1 (100)						

^a^minute; ^b^day; ^c^Dandong Yichuang Pharmaceutical Co.; ^d^only data available; ^e^hour.

ILN, Inguinal Lymph Nodes; SM, Submucosal through endoscopy; WS, Web Space; SBM, Small Bowel Mesentery; SC, Subcutaneous (groin); FJ, Feeding Jejunostomy.

## Discussion

The present systematic review provided a measurable opportunity to evaluate the current literature reporting on fluorescence-guided surgery to identify underlying similarities and divergent determinants of effectiveness for CL prevention during esophageal cancer resections. Indocyanine green is a tricarbocyanine iodide compound that was approved by the U.S. Food and Drug Administration in 1959 for medical purposes and has become extensively used over several clinical applications as a fluorescent diagnostic agent. Ultimately, the literature demonstrated large evidence of successful implementations in upper gastrointestinal surgery, particularly in the field of esophageal cancer care (26–28).

However, the increasing clinical experience in the ICG-assisted esophagectomy has been recently reported by other authors, who did not include the newest comparative analyses from high-volume series (6). Types of fluorescence-based guidance primarily integrated with esophagectomy include ICG-mediated angiography and lymphography, which provide a measure for visceral perfusion and clear visualization of the TD, respectively. Intraoperative ICG angiography has recently been established as a routine procedure that provides enhanced assessment of the integrity of the right gastroepiploic artery and tissue perfusion of the gastric conduit, to document adequate visceral state before fashioning the esophagogastric anastomosis (27). On the other hand, ICG-Lg has been developed to spread the fluorescent tracer over the lymphatic system, allowing the prompt identification of TD throughout the thoracoscopic stage of minimally invasive esophagectomy, especially showing its transdiaphragmatic course and the confluence of tributary or aberrant ducts. The expedited blood outflow and the pharmacokinetics of ICG allow angiographic and lymphographic techniques to be performed simultaneously, with no interference reported in any of the studies included in this systematic review. The administration of diluted concentrations of ICG has been reported via different routes, including the injection into the interdigital folds of the feet, the subcutaneous space, the inguinal lymph nodes, or the mesentery root of the small intestine. The findings from this systematic review demonstrated high proportions of accurate visualizations after every type of administration, assuming the rare failures of the technique to be due to independent anatomical features. Therefore, none of the reported ICG-Lg procedures demonstrated technical superiority or significant patient risk, allowing esophageal centers to adopt an individualized approach based on their routine surgical practice. No specific findings supported the definition of an optimal IGC concentration, as no evidence of defective TD visualization or dose-induced toxicity (median lethal dose, 50–80 mg/kg) was reported (29). It has to be stated that ICG-Lg procedures were cautiously performed in selected patients, while medical conditions were clinical reasons for exclusion from ICG administration (i.e., iodine allergy, pregnancy, preoperative liver dysfunction, any evidence of uncontrolled systemic diseases, or other inappropriate conditions to physicians). Eventually, the present systematic review provides the benefit of the concentration range of current administration (0.2-0.5 mg/kg or 2–25 mg), which represents an evidence-based recommendation of safety and clinical efficacy.

**Figure 3:**
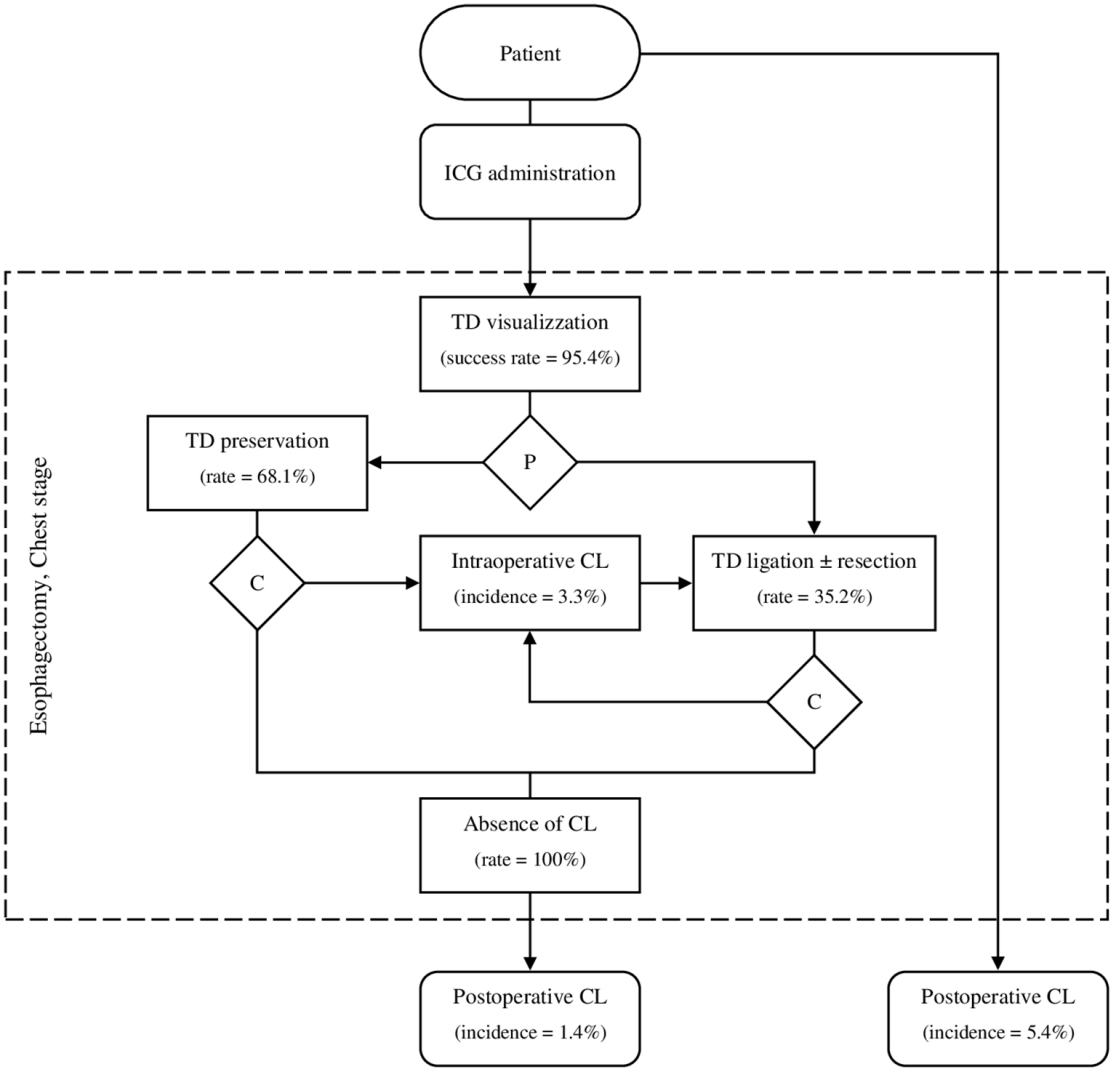
Procedural algorithm of ICG-Lg during esophageal cancer surgery. ICG, Indocyanine green; TD, Thoracic duct; P, Pre-emptive maneuver for chyle leak; C, Intraoperative check for immediate chyle leak; CL, Chyle leak.

The amphiphilic and dimensional characteristics (mass = 751.4 Da) of ICG tracer allow an extensive trans-diaphragmatic spread over the lymphatic system, entailing the clear visualization of TD and the associated variants of secondary tributaries (30, 31). The crucial point that emerges from the studies of this systematic review is the preventing strategy for CL (i.e., pre-emptive TD preservation or ligation), and the subsequent degree of surgical manipulation throughout the thoracoscopic phase of esophagectomy. Pre-emptive TD preservation appears to be the predominant technique of choice (68.1%) to minimize the occurrence of lymphatic injuries from surgical maneuvers. However, it has been reported that specific oncological, anatomical, or accidental/iatrogenic circumstances can occasionally force surgeons to TD handling or dissection (32). For this reason, an ultimate NIR inspection is commonly recommended to verify the integrity of lymphatic ducts after the mediastinal dissection of the lower esophagus, although no procedural guidelines have been shared to definitely exclude residual leaks. Conversely, other esophageal centers endorse the empirical belief that the pre-emptive TD ligation should be routinely performed, as the most accurate form of surgical control over the risk of iatrogenesis (33, 34). From the oncological standpoint, previous large-series studies demonstrated that TD dissection should not be legitimately considered among the targets of surgical resection for esophageal cancer, due to its rare tumor invasion and the low impact on survival (35, 36). However, some groups argue that the anatomical proximity between lymphatic ducts and the locoregional lymph nodes of the lower thoracic esophagus does not safely allow sparing TD during an extended mediastinal dissection. In accordance with the literature, the present systematic review reports significantly wider LNH after TD ligation and resection (OR 0.40, 95% CI -0.20 to 1.00, P<0.001), entailing higher chances for surgical removal of those lymph node stations which are more likely to be the site of locoregional metastases (**Figure 2**) (37). Eventually, the current ICG-assisted visualization of TD has allowed a highly heterogeneous management process (**Figure 3**), and no conclusive evidence has been supporting a specific surgical strategy.

CL is a severe postoperative complication that may entail acute respiratory failure or progressive physical deterioration, potentially leading to death (up to 17% of cases) (38). Although conservative treatment could avoid the need for further surgery, a successful resolution is uncertain and requires prolonged hospital stay and delayed chest drainage discontinuation. Despite improvements from applying IGC guidance for intraoperative CL identification, reoperation also demonstrated suboptimal success rates (80%) and a significant impact on patient functions and postoperative recovery (i.e., associated morbidity of 6–33%, and mortality of 0–33%) (39). Consequently, the occurrence of CL after esophagectomy potentially means the loss of eligibility for adjuvant therapy and the interruption of the multimodal sequence, preventing patients from undergoing optimal care. For this reason, the effective prevention strategy, early diagnosis, and standardized multidisciplinary management are fundamental requirements for such a deceptive surgical aspect, demanding high-level infrastructural resources and the selection of tertiary centers for esophageal cancer care.

Despite the methodological assumptions, this systematic review includes biases related to the structure and level of evidence of the selected articles. Further to the FDA approval and the experimental validation, intraoperative ICG-Lg for TD identification has been rapidly integrated into clinical practice and preliminary experience was reported in non-randomized observational studies. The quality assessment of this review (**Supplementary Material 3**) highlighted the moderate quality of the selected studies, which was exacerbated by the variety in ICG administration and surgical practice of each center. In addition to the ICG-Lg heterogeneity, the results of the present review were also impaired by the population size of individual studies, determining higher weighted relevance for larger series reports and their respective findings. Next-generation analysis should be designed to evaluate the efficacy of specific prevention strategies for CL, performed through ICG assistance and within uniform and controlled study settings.

## Conclusions

This review restrictively focused on a specific subject in esophageal cancer surgery, which demonstrated great effectiveness and procedural safety, with high potential for the development of ICG-Lg as a routine procedure to be performed throughout esophagectomy. However, the high accuracy and safety of intraoperative ICG-Lg did not associate with a standardized mitigation strategy for postoperative CL, generating inconclusive evidence on the effect of pre-emptive maneuvers and the subsequent extent of lymph node dissection. Although ICG-Lg significantly demonstrated to enhance anatomical landmarks for esophageal surgeons, the next randomized trials should be specifically designed to identify proper indications and mediastinal dissection in esophageal cancer surgery.

## Ospedale San Raffaele (OSR) Centro Cerca e Ricerca (CCeR) Collaborative group

Italy: Lavinia A. Barbieri, Riccardo Calef, Agnese Carresi, Andrea Cossu, Carolina Nardi, Floriana Iannace, Filippo Ostinelli, Lorenzo Rosi, Davide Socci, Elio Treppiedi, Stefano Turi (Department of Gastrointestinal Surgery, IRCCS San Raffaele Scientific Institute).

## Data Availability

The original contributions presented in the study are included in the article/**Supplementary Material**. Further inquiries can be directed to the corresponding author.
