# Supplementary material for: Indocyanine green-assisted lymphography for intraoperative chyle leak prevention during esophageal cancer surgery: a systematic review of the literature
Source: Front Oncol. 2026 Mar 3;16:1741834. doi: 10.3389/fonc.2026.1741834 (PMC12992011; doi:10.3389/fonc.2026.1741834)
Supplement: Supplementary file 2 [file Table2.docx]

**Supplementary material 2. Query strategy according to PICO guidelines.**

| **Engine** | **PICO** | **#** | **Searches** | **Results** |
| --- | --- | --- | --- | --- |
| PubMed | Population | 1 | ("esophageal neoplasms"[MeSH Terms] OR "esophagus"[MeSH Terms] OR "esophageal"[Title/Abstract] OR "esophagogastric"[Title/Abstract] OR "gastroesophag*"[Title/Abstract]) AND ("neoplas*"[Title/Abstract] OR "cancer*"[Title/Abstract] OR "carcino*"[Title/Abstract] OR "adenocarcino*"[Title/Abstract] OR "tumor"[Title/Abstract] OR "tumors"[Title/Abstract] OR "malign*"[Title/Abstract]) | 84727 |
|  |  | 2 | ("esophagectomy"[MeSH Terms] OR "esophagectom*"[Title/Abstract]) | 18709 |
|  | Intervention | 3 | ('indocyanine green'/exp OR 'fluorescence'/exp OR 'lymphography'/exp OR 'fluorescent dye':ti,ab OR 'indocyanine green':ti,ab OR fluorescen*:ti,ab OR lymphograph*:ti,ab) | 21198 |
|  | Comparison | 4 | *No comparison applies* | / |
|  | Outcome | 5 | ("thoracic duct"[MeSH Terms] OR "thoracic duct"[Title/Abstract]) | 6444 |
|  |  | 6 | ("chyle"[MeSH Terms] OR "chylothorax"[MeSH Terms] OR "chyl*"[Title/Abstract] OR "chyle leak*"[Title/Abstract] OR "lymphatic abnormalities"[MeSH Terms] OR "lymphatic diseases/surgery"[MeSH Terms] OR "lymph*"[Title/Abstract]) | 1154168 |
|  | Query | 7 | (#1 OR #2) AND (#3) AND (#5 OR #6) AND ("english"[Language] OR "italian"[Language]) AND (2020/01/01:3000/12/31[Date - Publication]) | 101 |

| **Engine** | **PICO** | **#** | **Searches** | **Results** |
| --- | --- | --- | --- | --- |
| Embase | Population | 1 | ('esophageal neoplasms'/exp OR 'esophagus'/exp OR esophageal:ti,ab OR esophagogastric:ti,ab OR gastroesophag*:ti,ab) AND (neoplas*:ti,ab OR cancer*:ti,ab OR carcino*:ti,ab OR adenocarcino*:ti,ab OR tumor:ti,ab OR tumors:ti,ab OR malign*:ti,ab) | 144075 |
|  |  | 2 | 'esophagectomy'/exp OR esophagectom*:ti,ab | 32234 |
|  | Intervention | 3 | 'indocyanine green'/exp OR 'fluorescence'/exp OR 'lymphography'/exp OR 'fluorescent dye':ti,ab OR 'indocyanine green':ti,ab OR fluorescen*:ti,ab OR lymphograph*:ti,ab | 971980 |
|  | Comparison | 4 | *No comparison applies* | / |
|  | Outcome | 5 | 'thoracic duct'/exp OR 'thoracic duct':ti,ab | 8029 |
|  |  | 6 | 'chyle'/exp OR 'chylothorax'/exp OR chyl*:ti,ab OR 'chyle leak*':ti,ab OR 'lymphatic abnormalities'/exp OR 'lymphatic diseases/surgery' OR lymph*:ti,ab | 1595635 |
|  | Query | 7 | (#1 OR #2) AND (#3) AND (#5 OR #6) AND (LANGUAGE(english) OR LANGUAGE(italian)) AND (PUBYEAR AFT 2019) AND ('article'/it OR 'review'/it) | 170 |

| **Engine** | **PICO** | **#** | **Searches** | **Results** |
| --- | --- | --- | --- | --- |
| Scopus | Population | 1 | ( INDEXTERMS ( "esophageal neoplasms" ) OR INDEXTERMS ( esophagus ) OR TITLE-ABS ( esophageal ) OR TITLE-ABS ( esophagogastric ) OR TITLE-ABS ( gastroesophag* ) ) AND ( TITLE-ABS ( neoplas* ) OR TITLE-ABS ( cancer* ) OR TITLE-ABS ( carcino* ) OR TITLE-ABS ( adenocarcino* ) OR TITLE-ABS ( tumor ) OR TITLE-ABS ( tumors ) OR TITLE-ABS ( malign* ) ) | 118927 |
|  |  | 2 | ( INDEXTERMS ( esophagectomy ) OR TITLE-ABS ( esophagectom* ) ) | 21763 |
|  | Intervention | 3 | ( INDEXTERMS ( "indocyanine green" ) OR INDEXTERMS ( fluorescence ) OR INDEXTERMS ( lymphography ) OR TITLE-ABS ( "fluorescent dye" ) OR TITLE-ABS ( "indocyanine green" ) OR TITLE-ABS ( fluorescen* ) OR TITLE-ABS ( lymphograph* ) ) | 1338070 |
|  | Comparison | 4 | *No comparison applies* | / |
|  | Outcome | 5 | ( INDEXTERMS ( "thoracic duct" ) OR TITLE-ABS ( "thoracic duct" ) ) | 7882 |
|  |  | 6 | ( INDEXTERMS ( chyle ) OR INDEXTERMS ( chylothorax ) OR TITLE-ABS ( chyl* ) OR TITLE-ABS ( "chyle leak*" ) OR INDEXTERMS ( "lymphatic abnormalities" ) OR INDEXTERMS ( "lymphatic diseases/surgery" ) OR TITLE-ABS ( lymph* ) ) | 1292616 |
|  | Query | 7 | (#1 OR #2) AND (#3) AND (#5 OR #6) AND ("english"[Language] OR “italian"[Language]) AND (2020/01/01:3000/12/31[Date - Publication]) | 153 |
